# The Chronotopic™ System for Pulsatile and Colonic Delivery of Active Molecules in the Era of Precision Medicine: Feasibility by 3D Printing via Fused Deposition Modeling (FDM)

**DOI:** 10.3390/pharmaceutics13050759

**Published:** 2021-05-20

**Authors:** Alice Melocchi, Marco Uboldi, Francesco Briatico-Vangosa, Saliha Moutaharrik, Matteo Cerea, Anastasia Foppoli, Alessandra Maroni, Luca Palugan, Lucia Zema, Andrea Gazzaniga

**Affiliations:** 1 Sezione di Tecnologia e Legislazione Farmaceutiche “M. E. Sangalli”, Dipartimento di Scienze Farmaceutiche, Università degli Studi di Milano, 20133 Milan, Italy; alice.melocchi@unimi.it (A.M.); marco.uboldi@unimi.it (M.U.); saliha.moutaharrik@unimi.it (S.M.); matteo.cerea@unimi.it (M.C.); anastasia.foppoli@unimi.it (A.F.); alessandra.maroni@unimi.it (A.M.); luca.palugan@unimi.it (L.P.); andrea.gazzaniga@unimi.it (A.G.); 2 Dipartimento di Chimica, Materiali e Ingegneria Chimica “G. Natta”, Politecnico di Milano, Piazza Leonardo da Vinci 32, 20133 Milan, Italy; francesco.briatico@polimi.it

**Keywords:** 3D printing, fused deposition modeling, multi-component systems, chronopharmaceutics, controlled release, pulsatile release, colon targeting

## Abstract

The pulsatile-release Chronotopic™ system was conceived of as a drug-containing core surrounded by a coat made of swellable/soluble hydrophilic polymers, the latter being able to provide a programmable lag phase prior to drug liberation. This system was also proposed in a colon-targeting configuration, entailing a gastroresistant film to prevent early interaction of the inner coat with gastric fluids and enabling the attainment of a lag phase matching the small intestinal transit time. Over the years, various multiple-step manufacturing processes have been tested for the fabrication of the Chronotopic™ system in both its configurations. This work focused on the evaluation of 3D printing by fused deposition modeling in view of its potential towards product personalization, on demand one-step manufacturing and efficient scale down of batches. The feasibility of each part of the Chronotopic™ system was independently investigated starting from in-house made filaments, characterizing the resulting specimens for physico-technological and performance characteristics. The printing parameters identified as suitable during the set-up phase were then used to fabricate prototypes either in a single step for the pulsatile configuration or following two different fabrication approaches for the colon-targeting one.

## 1. Introduction

Chronopharmaceutics relates to the development of drug delivery systems (DDSs) able to control the release of an active ingredient conveyed according to a body’s biological rhythms, having, as its ultimate goal, the improvement of compliance, efficacy and efficiency of pharmacological treatment [1,2]. These DDSs are generally designed to enable one or more lag phases of programmable duration prior to drug release, being thus referred to as pulsatile- or delayed-release systems [3,4]. There is a range of diseases characterized by onset and symptoms occurring late at night or on awakening (e.g., bronchial asthma, ischemic heart disease, atrial fibrillation, duodenal ulcer, sleep disorders, and rheumatoid arthritis) [5,6]. In the treatment of these pathologies, evening administration of drug products having a release pattern able to meet the relevant time of occurrence would limit risk or discomfort for the patient. Indeed, both unnecessary exposure to the drug induced by prolonged-release DDSs and sleep interruption for the administration of immediate-release dosage forms may be avoided. Oral pulsatile DDSs that provide lag phases matching the small intestine transit time, which has been demonstrated to be fairly consistent (3 ± 1 h SD), can also be used to target the colonic region [7,8,9,10,11]. However, these systems need to be provided with a gastroresistant film in order to overcome the variability of their residence time in the stomach. Colonic DDSs are highly beneficial for the treatment and prevention of local illnesses (e.g., inflammatory bowel disease, colorectal adenocarcinoma) or may improve the systemic absorption of active molecules having stability and permeability issues in the small intestine (e.g., proteins, peptides, nucleic acids) [12,13,14,15,16].

The Chronotopic™ system for pulsatile release was developed in the early 1990s as a solid core surrounded by a release-controlling coat made of swellable/soluble hydrophilic polymers [3,17]. Upon contact with biological fluids, such materials undergo a glass–rubber transition with the formation of a gel barrier, of which dissolution/erosion is responsible for a lag phase prior to release of the active molecules conveyed in the inner core. The lag time turned out to be programmable, both in vitro and in vivo, by changing the coating thickness and composition [18,19,20]. Over the years both single- (e.g., tablets, soft- and hard-gelatin capsules) and multiple-unit (e.g., pellets, granules, mini-tablets) dosage forms, characterized by either immediate or prolonged release patterns, were employed as inner cores [3,4,21,22]. In addition, a number of manufacturing techniques were tested for application of release-controlling coats onto solid cores. More recently, the reservoir system was upgraded by designing a functional container in the form of a capsule shell (Chronocap™) to be filled with different formulations and assembled after fabrication, even immediately before administration [23,24,25]. The manufacturing of capsular devices required to switch to hot-processing techniques, such as injection molding and fused deposition modeling (FDM) 3D printing, and to select thermoplastic polymeric formulations with suitable processability [26,27]. The possibility of applying an enteric coating to Chronotopic™ and Chronocap™ systems was also demonstrated in view of their possible use as colon-targeting DDSs [17,28,29].

The potential and cost-effectiveness of 3D printing in the manufacturing of personalized and innovative drug products, especially when small batches are involved, were largely confirmed in the last five years [30,31,32,33,34]. Great attention is currently being paid to versatility-oriented design strategies and scale-down fabrication opportunities, which could better suit the rising needs of precision medicine [35,36,37,38]. This involves tailoring the medical treatment to the peculiar characteristics of small groups of patients or even to those of each individual, and could include type, dose, administration mode, composition and release performance of the DDS under development. Considering its real-time prototyping capabilities and the chance to attain complex structures in a single, relatively simple production step, 3D printing is also expected to play a key role in the R&D areas of pharmaceutical industry in the coming years [39,40,41,42].

Based on these premises, the aim of the present work was to demonstrate the feasibility by FDM of the Chronotopic™ system, in both pulsatile and colonic release configurations, consistent with the needs of precision medicine. The development of formulations suitable for FDM based on pharmaceutical-grade polymers already approved for oral administration, rather than commercial plastic filaments, has already been investigated and described in previous manuscripts [43,44,45,46]. In this respect, the experience acquired was a fundamental starting point for this work. However, 3D printing of both the above mentioned configurations of the system using a dual-arm printer, which required to alternate two/three different formulations during fabrication, represented the new challenge here faced. In particular, the feasibility of different manufacturing approaches and the possibility of fine-tuning the release performance (e.g., duration of the lag phase, release profile of the drug-containing core) by changing virtual models, or formulation and printing parameters, were evaluated.

## 2. Materials and Methods

### 2.1. Materials

Hydroxypropyl cellulose, HPC (Klucel^®^ LF, Ashland, Kearny, NJ, USA); low viscosity hydroxypropyl cellulose, HPC SSL (Nisso HPC SSL, Nisso, Tokyo, Japan); methacrylic acid copolymer, EDR (Eudragit^®^ L 100-55, Evonik, Essen, Germany); polyvinyl alcohol, PVA (Gohsenol^®^ EG 03P, Mitsubishi Chemical, Tokyo, Japan); glycerol, GLY (Pharmagel, Milan, Italy); polyethylene glycol 400, PEG (Clariant Masterbatches, Milan, Italy); triethyl citrate, TEC (Sigma Aldrich, Milan, Italy); caffeine, CFF (ACEF, Milan, Italy); sodium starch glycolate, EXP (Explotab^®^ CLV, JRS Pharma, Rosenberg, Germany); high-amylose maize starch, AMY (Amylo^®^ N-460, Roquette Pharma, Lestrem, France); and polylactic acid filament (TreeD Filaments, Milan, Italy).

### 2.2. Methods

All materials, except for PEG, GLY and TEC, selected as plasticizers, were kept in an oven at 40 °C for 24 h prior to use. Plasticized polymeric formulations (Table 1) were prepared in a mortar by manually adding the selected plasticizer to the dry polymer, and then the other components, under continuous mixing with a pestle. The amount of plasticizer was expressed as % by weight on the polymer. 30% of EXP or AMY and 10% of CFF, expressed as % by weight on the plasticized polymer, were added if required.

#### 2.2.1. Extrusion of Filaments

Filaments were prepared by hot melt extrusion (HME) using a bench-top twin-screw extruder (Haake™ MiniLab II, Thermo Scientific, Madison, WI, USA) equipped with counter-rotating screws and a custom-made aluminum circular die (ø = 1.80 mm), as described before [47]. Extruded rods were manually pulled and forced to pass through a 1.80 mm caliper connected to the extruder. After production, filament diameter was verified every 5 cm in length and portions out of the acceptable diameter range (i.e., 1.75 ± 0.05 mm) were discarded. HME parameters are reported in Table 1 together with the torque registered during the extrusion of the investigated formulations.

The type and amount of plasticizers were adjusted, based on the torque values recorded, to enable continuous extrusion throughout the barrel of the employed extruder that had a limited length (12 cm). This would indeed result in relatively short-lasting exposure of the material to the temperature and shear stress conditions that cause its softening/melting. Moreover, the mechanical properties of the resulting filaments were found to impact also on effective printability of the different formulations. Depending on whether problems of filament rupturing or wrapping around the 3D printer gears were encountered, small increases or decreases in the amount of plasticizer (1%) were systematically attempted. This trial and error approach was pursued until formulations suitable for both extrusion of filaments and feeding of the printer were attained.

#### 2.2.2. Printing of Prototypes

FDM was performed by a Kloner3D 240^®^ Twin (Kloner3D, Florence, Italy) printer equipped with 0.5 mm nozzle. Computer-aided design (CAD) files were purposely prepared using Autodesk^®^ AutoCAD^®^ 2016 software version 14.0 (Autodesk Inc., San Rafael, CA, USA). Virtual models were saved in stl format and imported into the printer software (Simplify 3D, Milan, Italy). Details relevant to the CAD design and the printing of prototypes are reported in Section 3. In particular, drug-containing cores, release-controlling shells and multiple-component reservoir systems were prepared. Dumbbell specimens mainly intended for mechanical characterization were also produced.

#### 2.2.3. Characterization of Prototypes

Printed prototypes were checked for weight (analytical balance BP211, Sartorius, Göttingen, Germany; *n* = 6), height as well as diameter (digital caliper, Mitutoyo, Tokyo, Japan), thickness (MiniTest FH7200 equipped with FH4 probe, ø sphere = 1.5 mm, ElektroPhysik, Köln, Germany; *n* = 6), and relevant digital photographs were acquired (Hero 6, GoPro, Milan, Italy; Dino Lite Digital Microscope coupled with Dino Capture software, Dino-Lite, VWR International, Milan, Italy). Adhesion between adjacent layers and water tightness of the release controlling shells was evaluated as reported in [43]. Samples containing water sensitive paper strips, which are stained blue by aqueous droplets impinging on them, were immersed for 10 min in appropriate media (i.e., distilled water and phosphate buffer pH 6.8 for pulsatile-release and gastroresistant shells, respectively) kept at 37 ± 0.5 °C, withdrawn and cut into halves to check any change in color of the paper strips.

The release performance of drug-containing prototypes was then evaluated. To overcome sticking problems related to the formation of a gel layer around the pulsatile-release prototypes, a three-position USP38 disintegration apparatus (Sotax, Aesch, Switzerland) was used, according to a previously set up method [18]. Each basket-rack assembly moved at a 31 cycles/min rate in a separate vessel filled with 800 mL of water at 37 ± 0.5 °C. To make data comparable, uncoated cores and prototypes with enteric soluble coats were tested in the same apparatus and, only for the coated ones, under the conditions of the “Dissolution Test for Delayed-Release Dosage Forms” (Method B, USP38). Fluid samples were withdrawn at fixed time points and assayed spectrophotometrically (λ = 205 nm). By linear interpolation of the release data immediately before and after the time point of interest, the following parameters were determined: (i) time to 10% release (t_10%_), which was employed to define the lag phase prior to release; (ii) time to 90% release (t_90%_), which was used to calculate the time to complete the release as t_90%_–t_10%_, named pulse time; iii) time to 80% dissolution (t_80%_), which was selected to describe the performance of immediate release cores when tested as such. When an enteric soluble part was involved (i.e., gastroresistant shells, Chronotopic™ system in the colon-targeting configuration), release parameters in the phosphate buffer medium were calculated by subtracting 120 min to t_10%_ and t_90%_, which corresponded to the time elapsed in acidic medium.

#### 2.2.4. Mechanical Characterization

Mechanical analysis was carried out using an Instron 1185-5800R dynamometer (Instron, Glenview, IL, USA) equipped with a 10 kN load cell. Tensile tests were performed on 200 mm-long extruded filaments (*n* = 3). The initial clamping distance (L_0_) was approximately 130 mm while the crosshead speed was set at 1.25 mm/min. By measuring the applied displacement (ΔL) and the relevant load (F), strain (ε = ΔL/L_0_) and stress (σ = F/(πR^2^)) were calculated. From the stress–strain curves, the Young modulus (E), intended as the slope of the first linear part of the curve, was determined. Moreover, the peak or maximum stress (i.e., the value of stress obtained at the first strain beyond which such a parameter started to decrease) was selected as an index of the material strength (σ*). PLA commercial filament was employed as a reference material.

Further tensile tests involving the same experimental conditions were performed on dumbbell specimens (*n* = 3) having a gauge length of 68 mm and a cross-section of approximately 7.2 × 1.2 mm^2^ printed by setting an infill percentage of either 100% or 50%. The strain was evaluated as before described for filaments, while an apparent stress (σ_app_) was calculated assuming that the area of the cross-section was completely filled. From the σ_app_-ε curves, apparent modulus (E_app_) and apparent strength (σ*_app_) were estimated as discussed above.

## 3. Results and Discussion

### 3.1. Design Concept

The Chronotopic™ system in the pulsatile-release configuration consists in a drug-containing core surrounded by a polymeric barrier able to defer its interaction with aqueous fluids. Being composed of swellable/soluble hydrophilic polymers, this soluble/erodible coat, upon interaction with aqueous media, enables the formation of a gel layer that undergoes slow dissolution/erosion. The colon-targeting configuration was provided with a further external gastroresistant coat made of enteric-soluble polymers. With the aim of fabricating this system by FDM, a printer equipped with two independent arms able to work simultaneously or alternately, with diverse materials and under different process conditions, was employed. 3D printed items are fabricated through deposition of subsequent layers, which can be made of the same or of different materials/formulations. When adjacent parts having different composition are layered in the same object, a so-called multiple-composition structure is achieved. The dual arm equipment allows to increase the efficiency of production while overcoming cross-contamination and stability issues involved by the use of nozzles mounted on a same arm. In fact, when a printing head is activated, the inactive one moves to the side of the build plate, thus avoiding any leakage of undesired material on the item under fabrication. Moreover, because the printing heads are separated from each other, it would be possible to work with different formulations, also requiring diverse printing temperatures, thus possibly broadening the combinations of materials to be employed.

**Figure 1:**
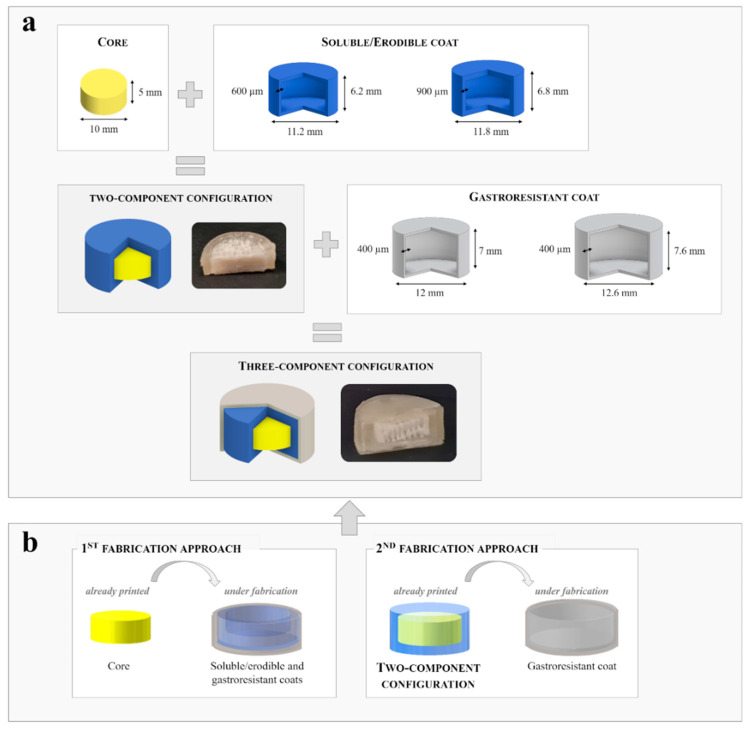
(**a**) Outlines and photographs of the configurations of the Chronotopic™ system including CAD files with dimensional details of the parts to be combined and (**b**) outline of the fabrication approaches envisaged for printing the three-component configuration.

The design conceived for the fabrication of the Chronotopic™ system in the above described configurations comprised a central part (i.e., core) and either one or two concentric coats, for pulsatile- and colonic-release, respectively (Figure 1a). The Chronotopic™ system in the pulsatile-release configuration, envisaging only one soluble/erodible single coating, would be printed as a two-component item. On the other hand, dealing with its colon-targeting configuration, which presents an additional gastroresistant external coating, it would be fabricated as a three-component structure. In particular:

- the core part could be either a drug-containing unit fabricated by FDM or any formulation fed during fabrication (e.g., powders, pellets, gels);
- the soluble/erodible coat was based on a swellable/soluble hydrophilic polymer (i.e., HPC);
- the gastroresistant coat was based on an enteric-soluble polymer (i.e., EDR).

These polymers were selected based on the experience gained in the development of formulations to be used for printing capsular devices [44,47].

CAD files were developed for each part. The core was designed as a cylinder, while the coatings were conceived as closed concentric hollow units of equal shape (i.e., shells) having different thickness and consequently outer height and diameter. In particular, two nominal thickness values were selected for the soluble/erodible coat (i.e., 600 and 900 µm), in order to investigate its ability to provide different lag times prior to release. On the other hand, the external gastroresistant coat was characterized by a nominal thickness of 400 µm and its height and diameter were adapted to match the dimensions of the underlying parts.

In view of the resolution limits already highlighted for the FDM technique, especially when dealing with coated and hollow systems characterized by the presence of small details, the feasibility of each part was independently evaluated in the set up phase (see Section 3.2) [48,49].

Using a dual arm printer, either one or two formulations at a time could be processed enabling the manufacturing of (i) two samples of the same composition, thus improving the rate of production, and of (ii) a two-component system by alternating the deposition of different formulations (i.e., combo mode). In this respect, the Chronotopic™ in the pulsatile-release configuration, being a two-component unit, was fabricated printing the drug-containing core with one printer head and using the other to concomitantly build up the HPC barrier around it. For the manufacturing of the system in the three-component colon-targeting configuration, two fabrication approaches were envisaged (Figure 1b). Both of them entailed the insertion of a previously printed unit into a growing shell by pausing the relevant printing process at a specific height and resuming it after the deposition of the unit itself. In particular, a printed drug-containing core would be inserted into a two-component shell made of HPC and EDR (1st fabrication approach), and a printed two-component unit, i.e., consisting of a core and a soluble/erodible coat, would be inserted into a single gastroresistant EDR-based shell (2nd fabrication approach). In the former case, not only a printed drug-containing core but also powder, pellets/granules or liquid formulations may be considered, which could be deposited inside the hollow part under fabrication.

### 3.2. Set up of the Printing Process

The main focus of the set up work was to improve the process and reproducibility of the product characteristics, with particular attention to the thickness of the release-controlling coats (i.e., adherence of the actual dimensional details to those designed). Based on recent literature relevant to the manufacturing of hollow systems by FDM, a systematic approach already proposed in few engineering works for setting the printing process was adapted to the fabrication of the Chronotopic™ system [50,51,52,53].

Simplify 3D printing software, enabling the operator to independently set a variety of parameters, was employed to evaluate the feasibility of each part conceived in the design concept: both the release-controlling coats, i.e., the soluble/erodible and gastroresistant ones, and the core. This approach would especially be useful for identifying and solving critical issues related to the single parts of the system. In this respect, the physico-technological characteristics of individual parts could hardly be evaluated dealing with the complete system fabricated in a single step, in view of the risk of damaging it and altering the features of each single component.

Further on, the individual settings identified for each part would be employed for printing the Chronotopic™ system in the different configurations involving the combo mode.

#### 3.2.1. Release-Controlling Coats

The release-controlling coats were designed as closed cylindrical hollow units, also referred to as shells, entailing the possibility to be manually filled. Feasibility of FDM for their fabrication starting from filaments made of HPC and EDR formulations was defined based on the independent measurements of weight and dimensions (i.e., height, diameter and thickness) of the printed shells. These were selected as critical quality parameters because they were already demonstrated to be fundamental for the overall performance of the Chronotopic™ system and, being their dimensional tolerances in the micrometric range, were challenging to be fabricated by FDM. This task was undertaken through fabrication of one part at a time to also reduce inter-batch variability and improve the consistency of subsequent prints.

The mechanical characteristics of the starting filaments were taken into account, as their stiffness and brittleness have to be tuned to allow their feeding into the equipment without breaking it. From the comparison with a standard PLA filament, taken as a reference, both the considered materials showed lower E and σ* [45] This did not impair filament printability and even reduced the risk of breakage following loading between the printer gears.

Preliminary trials were useful for the identification of the software parameters mainly affecting the quality of the shells:

- temperature of the build plate and of the nozzle, which would determine the adhesion of the first layer to the build plate and that of successive layers between each other, thus impacting on the possibility of attaining a unitary item;
- number and height of deposited layers, which would impact on the dimensions (e.g., height, diameter, thickness of the coats) and resolution of the final system;
- flow rate, i.e., amount of material deposited over time, which would determine the sample weight;
- cooling rate, which is controlled by setting the speed of the cooling fan, and layer at which the selected rate was applied (activation layer), which overall would affect adhesion between successive layers;
- printing speed, which not only would directly determine the printing time but also may affect material adhesion.

Empty units were manufactured in a continuous process to check their weight and external dimensions (i.e., diameter and height). In a second fabrication mode, printing of the shell was interrupted at a specific height from the bottom to enable manual filling of the cavity, either with water-sensitive paper strips or with a powder tracer. Then the process was restarted to complete the top surface of the device and close it. This allowed to study water tightness and release performance of the prototypes, respectively. During the process, open uncompleted shells were also sampled to enable thickness measurements of their base and lateral wall.

Based on the part design, thickness values would be in principle controlled by a range of interdependent parameters, which may show diverse impact depending on the direction of deposition. In the case of the cylinder bases, the layer thickness was determined along the z axis, which was the growing direction, and would depend on the expansion or contraction of the material and on a few other settings such as (i) printer nozzle diameter, (ii) layer height and (iii) number of layers deposited one on the top of the other. On the contrary, as the wall thickness was defined in the xy direction, it would be a function of the number of concentric layers deposited side by side and their possible overlapping.

**Figure 2:**
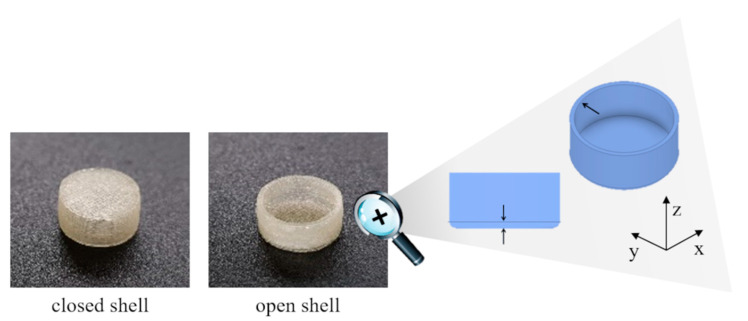
Photographs of empty closed and uncompleted open shells, and outline of the open shell highlighting details relevant to the thickness measurements.

In Figure 2 photographs of the printed prototypes and an outline showing the details relevant to the thickness measurements and the system of reference are reported.

Starting from the CAD file of each shell, samples were printed (i) using standard operating conditions provided by the software upon setting the high-resolution printing mode, and (ii) changing the process parameters previously identified as critical. In this respect, a trial and error approach aimed at minimizing the difference between actual and nominal thickness values was pursued. Secondly, thickness consistency in different parts of the shell (i.e., base and wall) was also considered. One printing condition at a time was modified, and then the resulting prototypes were characterized for the above-mentioned critical quality parameters to assess the outcome of the modifications implemented. For each polymeric formulation and type of shell a set of fine-tuned parameters was therefore identified. In order to close the shell, the use of the bridging parameter was essential, leading to the effective printing of top surfaces supported by only two vertical structures at their edges. This was shown possible, both when performing the continuous deposition for the fabrication of empty shells and when restarting after loading of the power tracer.

When working with the HPC filament, except for nozzle and build plate temperature, which were set at 180 and 50 °C, respectively, the tuned operating conditions turned out to be different for shells having diverse nominal thickness. The 600 µm thick HPC shells were successfully printed using a 120% flow rate, 40 mm/s printing speed and setting 2 layers of nominally 0.2 mm for the fabrication of the base. Conversely, 900 µm thick prototypes required a 100% flow rate and 4 layers of 0.2 mm for the cylinder bases.

For the EDR-based shells, the final operating conditions selected were: 195 and 100 °C for nozzle and build plate temperature, respectively; 80% flow rate; 7 mm/s printing speed; 4 layers of 0.150 mm for printing the base. In particular, the increase in layer number, the reduced layer height and the very low printing speed were required to avoid layer detachment and promote adhesion of the material.

In Table 2 and Table 3, the characteristics of HPC- and EDR-based shells, printed under standard and fine-tuned conditions, are summarized. By way of example, data relevant to EDR-based shell designed to match the Chronotopic™ system with a 600 µm thick swellable/erodible layer were reported.

In order to highlight resolution limits of the process, the difference between measured dimensions and relevant nominal values provided by the electronic model was also calculated (Δ parameters).

Overall, weight variation data (CV) were confirmed to be correlated to the dimensions of the printed samples, thus pointing out a certain constancy of the density of the material. The low reproducibility obtained setting standard printing conditions was improved by the fine-tuning approach. Comparing the external diameter of the printed items with the nominal values, a tendency of HPC to expansion and, conversely, of EDR to contraction and collapse after layer deposition was hypothesized. However, these phenomena could also be attributed to the capability of the equipment in use to execute commands, especially when involving fine movements for the fabrication of details of limited size. Indeed, resolution limits in 3D movements are well-known for desktop FDM printers and were attributed to the design, assembly and construction materials [49].

Overall, the thickness variability was lower for the cylinder bases with respect to the relevant wall, so that the equipment accuracy was confirmed to be higher for movements in the z axis than in the xy plane. Limited resolution was also confirmed by the Δ values. In this case, the reduction in thickness (i.e., from 900 to 400 µm) showed a worsening in precision. However, it turned out evident that the deviation of dimensions and thickness from the nominal values could be largely decreased by adjusting the printing settings. Indeed, fine-tuning of process conditions enabled to attain thickness values, in both wall and base areas, closer to the desired ones, independent of the composition and nominal thickness of the shell. Moreover, the data reproducibility was improved.

Using the fine-tuned parameters, shells filled with water sensitive paper strips and a drug tracer were also produced. Fabrication was interrupted at a specific height to enable manual filling of the cavity and then started again to ensure proper closure of the systems. A curing process was applied to few filled shells, by keeping them in an oven at 40 °C for 3 h because this could favor relaxation of internal stresses, which could result in the enhancement of layer-to-layer adhesion and relevant stability. Uncontrolled penetration of aqueous fluids trough adjacent layers not completely sealed was preliminary excluded testing shells filled with water sensitive paper strips. This was confirmed for both types of shell, independent of the curing step.

**Figure 3:**
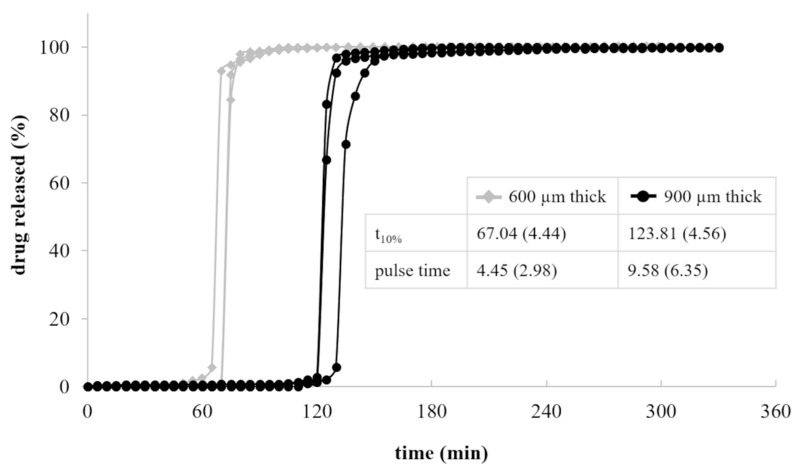
Release profiles of 600 and 900 µm thick HPC-based shells (average release parameters and relevant CV, in brackets, are listed in boxes).

In Figure 3, the release profiles of HPC-based shells having a nominal 600 and 900 µm thickness and filled with a powder tracer are reported.

As expected, upon immersion in aqueous fluids, the formation of a gel layer around the HPC-based shells was observed. The gel underwent slow dissolution/erosion phenomena, which hindered the penetration of the solvent into the cavity and deferred the liberation of the drug tracer. Lag time (i.e., t_10%_) and duration of release (i.e., pulse time) turned out to be dependent on the shell thickness. Indeed, increasing it from 600 to 900 µm, the lag time was extended of approximately 50 min. The pulse time was in both cases less than 10 min, pointing out that, once the first opening of the shell occurred, the thickness of the polymeric barrier to be dissolved/eroded would not mainly affect the time needed to complete the liberation of the conveyed formulation.

**Figure 4:**
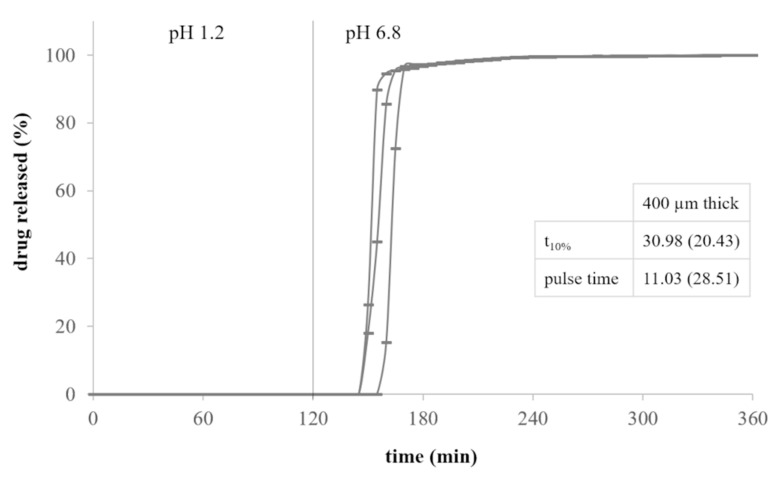
Release profiles of 400 µm thick EDR-based shells (average release parameters and relevant CV, in brackets, are listed in boxes).

Gastroresistant shells filled with the drug tracer showed the ability to withstand the acidic environment and to promptly release their content within approximately 30 min from the medium pH change (Figure 4). Indeed, the EDR-based prototypes remained intact and unchanged in the acidic medium and started to dissolve only when in contact with phosphate buffer. As a further demonstration that these structures were watertight, no release occurred in the first 2 h of testing.

For both the types of shell under investigation, the curing step was demonstrated not to modify the release performance.

#### 3.2.2. Core

The core of the Chronotopic™ system was designed to provide an immediate release of the active molecule conveyed after the lag phase and formulations for its manufacturing by FDM were accordingly selected. In particular, thermoplastic soluble polymers already employed for hot-processing with rather low molecular weight (i.e., PVA and HPC SSL) were evaluated for manufacturing of filaments via HME [54]. CFF was chosen as the drug tracer based on previous experience [55]. Plasticizers were added so that processing temperatures allowed the powder tracer to remain suspended in the filament without undergoing degradation phenomena (CFF melting and degradation temperature around 230 and 285 °C, respectively). A formulation strategy entailing the use of adjuvants to improve the dissolution rate of the cores was also pursued. EXP was selected as a disintegrant based on previously reported studies focused on the efficacy of a range of excipients in speeding up the dissolution rate from devices obtained by hot-processing techniques [54,56]. Moreover, AMY was included in the PVA-based formulation in view of literature references and preliminary data collected showing the ability of such an additive to improve the dissolution/erosion performance of molded polymeric barriers [57,58]. Formulation and HME parameters were defined to attain a continuous extrusion and mainly taking into consideration the printability and mechanical properties of the extruded filaments (Table 4).

All the considered filaments showed lower mechanical properties, especially in terms of E, with respect to the PLA reference. This was attributed to the presence of plasticizers, but it did not limit 3D printability while reducing the processing temperature. Filament printability was evaluated in terms of breaking, tendency to wrap around the printer gears, and quality of printed items (e.g., presence of visible defects, reproducibility in terms of weight, dimension and infill pattern). Interestingly, the addition of adjuvants had a favorable effect on E of both PVA- and HPC SSL-based filaments, without affecting their σ*. This suggested a good dispersion of the additive particles into the polymeric matrices, which is expected to be beneficial for the performance of the printed items.

Dumbbell specimens were selected for investigating also the influence of formulation and process parameters on the mechanical properties of printed parts. The infill percentage was modified and top and bottom layers were removed in order to obtain printed samples with potentially different porosity. Indeed, an infill below 50% in the absence of top and bottom layers resulted in items with too poor handling. For PVA-based samples 180 and 50 °C were set as nozzle and build plate temperature, respectively. Moreover, 0.1 mm layer height, 100% flow rate, 30 mm/s printing speed were employed. On the other hand, printing of HPC SSL-prototypes required to reduce the temperature of the nozzle to 150 °C, while that of the build plate was kept at 50 °C. The printing speed was set at 25 mm/s and flow rate at 120%, whereas the layer height was maintained at 0.1 mm. Considering dumbbell specimens printed with 100% infill, E_app_ values generally turned out comparable to E values relevant to filaments of the same composition (Table 5). This suggested that, by setting such an infill, it was possible to attain completely filled items, without unexpected cavities or voids. The results also confirmed that the FDM process had no major effect on the properties of the starting materials. As for σ*_app_, the data collected from dumbbell specimens resulted comparable with σ measured for the corresponding filaments and in some cases even higher. This could be ascribed to lower variability and smaller or less defects of printed samples with respect to manually-pulled extruded filaments.

Changes in the infill percentage caused a reduction of about a factor of 2 in E_app_, consistent with the infill reduction, which was halved. This confirmed that voids in the bulk of the material were increased in such a way that even the effective cross-section area was halved. On the other hand, σ*_app_ was even more decreased, probably because the voids in the cross-section acted as defects, which caused premature fracture of the sample during the test.

Based on the formulation and processing parameters tuned, cores with no top and bottom layers were printed according to the relevant CAD file (see Figure 1a).

**Figure 5:**
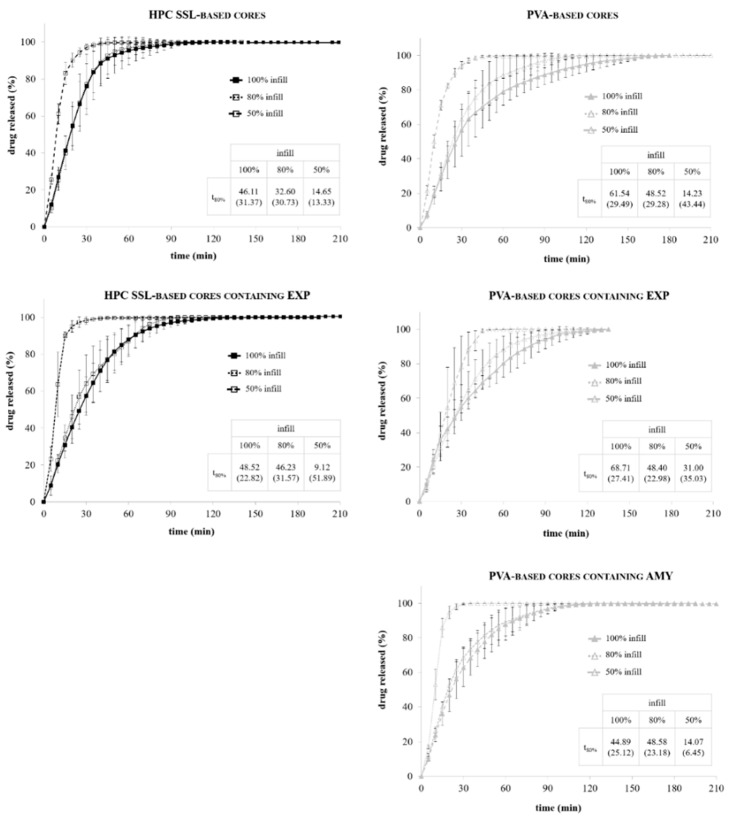
Release profiles of HPC SSL- and PVA-based cores (average release parameters and relevant CV, in brackets, are listed in boxes).

In Table 6 and Figure 5, physico-technological characteristics and release performance of HPC SSL- and PVA-based cores, with and without adjuvants, printed with different infill percentage are reported. Notably, the amount of the active ingredient conveyed in the cores was in all cases > of 98% with respect to the theoretical value calculated based on prototypes weight and composition.

In agreement with literature data, the removal of the top and bottom layers and the infill reduction turned out essential for speeding up the liberation of the drug tracer from the printed cores, probably due to the impact of such parameters on porosity and area exposed to the aqueous medium [58,59,60,61,62,63]. With all the formulations, the best results were obtained by decreasing the infill to 50%, independent of the presence of an adjuvant in the formulation. On the contrary, minimal differences in terms of t_80%_ were obtained between samples having 100 and 80% infill, which was attributed to a quite similar effective porosity of the structure. Indeed, purposely printed units were prepared interrupting fabrication at different heights to enable microscopic inspection inside the structure. While the infill pattern that could be seen inside the 50% infill structures was clearly rare, no remarkable differences were observed between the 100 and 80% ones. Once again, this could be attributed to the resolution limits of the printer and of the FDM technique, especially when dealing with micrometric tolerances. Moreover, as the equipment was designed and built up to execute the software commands with PLA filament, resolution might be even worse in view of the filament formulations employed.

When EXP was added, a tendency of the core to disintegrate, thus improving drug liberation rate, was only noticed in the case of samples composed of the polymer characterized by a lower viscosity (HPC SSL) and having the less coherent structure associated with the 50% infill. In the case of PVA-based cores, which showed the slower dissolution rate, the presence of AMY had a positive impact on the liberation rate, especially when dealing with samples printed with 50% infill. This was in agreement with preliminary literature findings regarding food packaging applications [64,65,66,67].

### 3.3. Chronotopic™ Prototypes for Pulsatile-Release and Colon-Targeting

Taking advantage of the set up work above described, the fabrication by FDM of the Chronotopic™ system in the pulsatile- and colonic-delivery configurations was then approached. The fine-tuned formulation and process parameters independently identified for the core and release controlling coats were set for operating the printer in the combo mode, according to the fabrication approaches described in Figure 1b. In particular, the printing process of the pulsatile-release system entailed the concomitant fabrication of core and soluble/erodible coating. For the colon-targeting configuration, either the core and the soluble/erodible coating or the two release-controlling HPC- and EDR-based coats were fabricated in the combo mode. In all cases, the printing process was interrupted to manually introduce previously printed parts (i.e., either the drug-containing core or the two-component pulsatile-release system) into the shell structure under fabrication (see Figure 1b) All the printing processes were successful with no need for adjusting parameters nor evidence of discontinuity/lack of adhesion of layers of different composition or printed after an interval. Moreover, data relevant to weight and external dimensions turned out consistent in terms of measurements and variability with those tuned in the set up phase.

**Figure 6:**
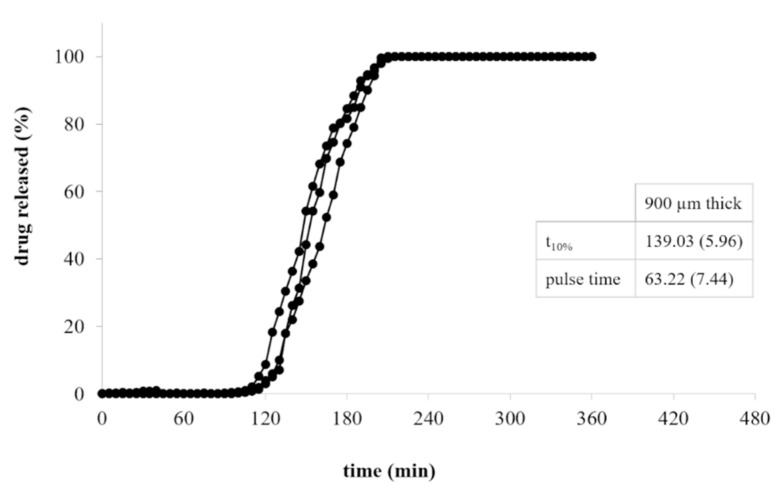
Release profiles of two-component systems composed of a PVA-based core containing AMY and printed with 50% infill and a 900 µm thick HPC coat (average release parameters and relevant CV, in brackets, are listed in boxes).

**Figure 7:**
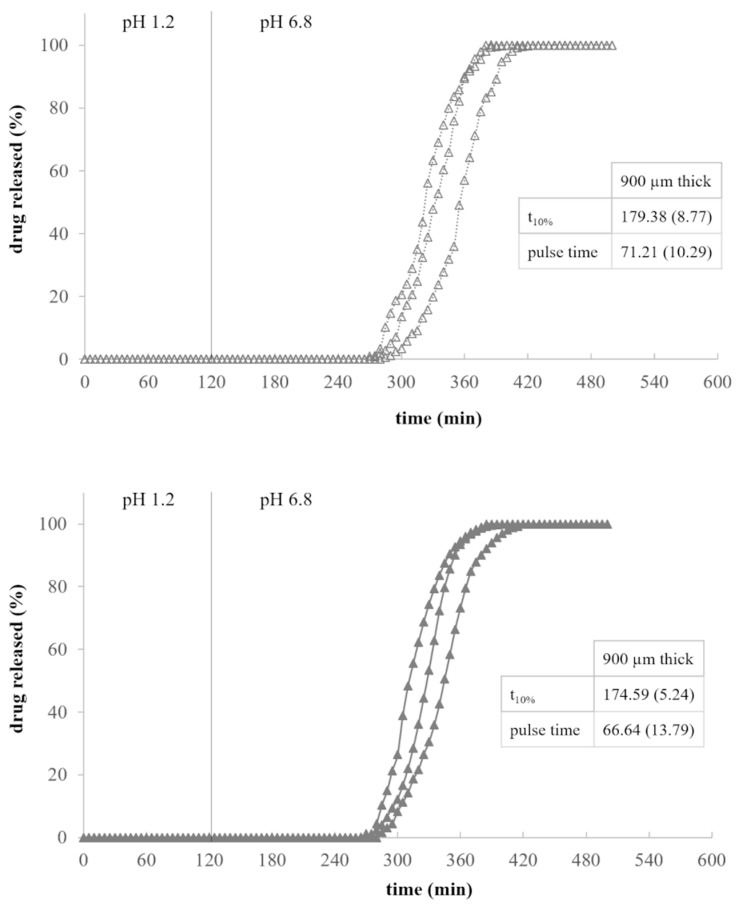
Release profiles of three-component systems fabricated following two different fabrication approaches and composed of a PVA-based core containing AMY and printed with 50% infill, a 900 µm thick HPC-based and a 400 µm thick EDR-based coats (average release parameters and relevant CV, in brackets, are listed in boxes).

By way of example, the release profiles of a two-component system for pulsatile release based on AMY-containing PVA cores fabricated using 50% infill and coated with a 900 µm thick HPC layer are reported in Figure 6. Furthermore, the performance of three-component systems of analogous composition with the addition of an external EDR-based coat, prepared according to the two different fabrication approaches conceived, are compared in Figure 7.

As described above for HPC-based shells, also in the case of the two-component systems the formation of an external gel layer deferred the exposure of the drug-containing core to the fluid. Considering the three-component devices, only after the dissolution of the external coat based on EDR, the pulsatile-release one could interact with the aqueous media and the above mentioned behavior could be activated. Overall, the lag phase prior to release obtained with all the systems was consistent with the performance shown by the single parts (i.e., drug-containing core and polymeric shells) when tested independently. The increase of the lag time, and more markedly that of the pulse time, was expected due to the longer time needed for dissolution of the drug from the printed core units with respect to that of CFF conveyed in the shells as a powder. More into detail, the time taken for dissolution was largely affected by the presence of one (Figure 6) or even two (Figure 7) adjacent coats covering the core and slowing down the water uptake. Taking advantage of this peculiar behavior, the thickness of the of the soluble/erodible coat could even be decreased. Three-component systems obtained according to different fabrication approaches pointed out very similar release performance, showing the ability to withstand in the acidic environment and to release their content after pH change. The overlap of the release profiles also confirmed the effective adhesion between layers of different composition when fabricated in the combo mode, regardless of whether the core and the pulsatile-release coat or the two release-controlling coatings were concerned.

## 4. Conclusions

The Chronotopic™ is a reservoir system that has shown potential, both in vitro and in vivo, for pulsatile release and colon targeting based on a time-dependent approach. The main therapeutic objectives pursued so far with the Chronotopic™ system were the therapy of chronopathologies characterized by recurrent symptoms in the late night/early morning hours and the treatment of local diseases of the colonic region. However, the need for oral administration of biological drugs and the rising interest in precision medicine could represent a decisive incentive for the development of personalized configurations of this system. Therefore, the possibility of exploiting additive manufacturing for its small-scale production was considered, which would be versatile in terms of dose, drug-containing formulations and achievable release performance. In the present work, the design concept and fabrication approach for 3D printing via FDM of multi-component systems entailing a core and two release controlling coats were first presented, and the feasibility of printing two-component and three-component prototypes for pulsatile and colonic release, respectively, was systematically assessed and demonstrated.

## Figures and Tables

**Table 1:** Formulation of filaments and extrusion parameters.

Formulation	T (°C)	Screw Speed (rpm)	Torque (N·cm)
HPC	160	80	50
EDR + 25% TEC	165	80	120
(PVA + 15% GLY) + 10% CFF	185	70	110
(PVA + 15% GLY) + 10% CFF + 30% EXP	190	70	150
(PVA + 15% GLY) + 10% CFF + 30% AMY	190	70	140
(HPC SSL + 5% PEG 400) + 10% CFF	160	100	45
(HPC SSL + 5% PEG 400) + 10% CFF + 30% EXP	160	100	65

**Table 2:** Weight and dimension characteristics of HPC-based shells.

			Average	CV	Average Δ with Respect to the Electronic Model (%)
600 µm—thick shells	Standard conditions	Weight (mg)	128.12	9.23	
		External diameter (mm)	10.38	3.89	−7.32
		Height (mm)	6.78	2.41	+9.35
		Base thickness (µm)	504	6	−16.00
		Wall thickness (µm)	448	8	−25.33
	Fine-tuned conditions	Weight (mg)	136.57	8.02	
		External diameter (mm)	11.33	1.82	+1.16
		Height (mm)	6.14	1.65	+0.97
		Base thickness (µm)	615	6	+2.50
		Wall thickness (µm)	572	7	−4.67
900 µm—thick shells	Standard conditions	Weight (mg)	201.13	12.55	
		External diameter (mm)	10.85	3.87	−3.13
		Height (mm)	7.24	3.22	+6.47
		Base thickness (µm)	566	11	−37.11
		Wall thickness (µm)	1032	7	+14.67
	Fine-tuned conditions	Weight (mg)	187.81	7.14	
		External diameter (mm)	11.88	1.02	+0.68
		Height (mm)	6.91	1.35	+1.62
		Base thickness (µm)	897	4	+0.33
		Wall thickness (µm)	900	4	+0.00

**Table 3:** Weight and dimension characteristics of EDR-based shells.

			Average	CV	Average Δ with Respect to the Electronic Model (%)
400 µm—thick coatings	Standard conditions	Weight (mg)	79.12	11.98	
		External diameter (mm)	11.00	7.45	−8.33
		Height (mm)	7.45	2.98	+6.43
		Base thickness (µm)	563	14	+40.75
		Wall thickness (µm)	459	10	+14.75
	Fine-tuned conditions	Weight (mg)	76.0	7.0	
		External diameter (mm)	12.15	2.10	−1.25
		Height (mm)	7.13	1.64	+1.85
		Base thickness (µm)	398	6	−2.25
		Wall thickness (µm)	423	8	+5.75

**Table 4:** E and σ* of filaments produced by HME and of commercially available PLA filament.

Formulation	E, GPa (s.d.)	σ*, MPa (s.d.)
(PVA + 15% GLY) + 10% CFF	0.41 (0.01)	16.5 (0.70)
(PVA + 15% GLY) + 10% CFF + 30% EXP	0.94 (0.09)	13.8 (1.20)
(PVA + 15% GLY) + 10% CFF + 30% AMY	1.29 (0.22)	14.8 (5.30)
(HPC SSL + 5% PEG 400) + 10% CFF	0.33 (0.08)	3.6 (0.90)
(HPC SSL + 5% PEG 400) + 10% CFF + 30% EXP	0.81 (0.17)	3.2 (1.00)
Commercial PLA filament	2.88 (0.09)	43.4 (2.80)

**Table 5:** E_app_ and σ*_app_ of 3D printed dumbbell specimens.

	Infill 100%		Infill 50%	
Formulation	E_app_, GPa (s.d.)	σ*_app,_ MPa (s.d.)	E_app,_ GPa (s.d.)	σ*_app_, MPa (s.d.)
(PVA+15% GLY) + 10% CFF	0.40 (0.05)	15.5 (0.20)	0.20 (0.10)	5.5 (0.10)
(PVA+15% GLY) + 10% CFF + 30% AMY	1.25 (0.03)	20.7 (0.40)	1.15 (0.06)	13.2 (0.90)
(PVA+15% GLY) + 10% CFF + 30% EXP	1.15 (0.06) a	15.1 (0.90)	0.64 (0.11)	6.3 (0.50)
HPC SSL + 5% PEG 400) + 10% CFF	0.53 (0.05)	6.6 (0.90)	0.22 (0.08)	1.8 (0.18)
(HPC SSL + 5% PEG 400) + 10% CFF + 30% EXP	0.29 (0.03)	3.0 (0.60)	0.06 (0.01)	0.6 (0.20)

**Table 6:** Weight and dimension characteristics of HPC SSL- and PVA-based cores printed with different infill percentages.

Formulation	(HPC SSL + 5% PEG 400) + 10% CFF						(PVA + 15% GLY) + 10% CFF								
	/			+30% EXP			/			+30% EXP			+30% AMY		
Infill	100%	80%	50%	100%	80%	50%	100%	80%	50%	1005	80%	50%	100%	80%	50%
Weight, mg (CV)	401.07 (6.61)	389.84 (6.96)	301.88 (7.84)	464.81 (11.55)	416.75 (10.42)	351.78 (11.83)	486.62 (2.92)	433.73 (3.06)	351.38 (8.019)	434.95 (2.34)	467.06 (5.95)	326.89 (6.89)	418.51 (1.01)	486.90 (4.12)	302.52 (5.11)
Height, mm (CV)	5.68 (0.99)	5.20 (1.90)	5.55 (1.99)	5.28 (2.33)	5.61 (3.97)	5.21 (4.93)	5.67 (1.73)	4.99 (2.18)	5.15 (3.96)	5.33 (2.35)	5.17 (3.38)	5.06 (2.24)	5.32 (1.68)	5.45 (2.20)	5.14 (6.55)
Diameter, mm (CV)	9.89 (1.40)	9.98 (1.74)	9.99 (3.51)	10.06 (1.30)	10.18 (1.43)	10.08 (1.88)	10.31 (1.28)	10.32 (1.30))	10.28 (1.90)	9.83 (0.78)	9.43 (1.55)	9.84 (2.02)	9.88 (1.17)	10.00 (1.06)	9.93 (3.02)

## Data Availability

The data presented in this study are available on request from the corresponding author.
